# The association of N-terminal pro-brain-type natriuretic peptide with hemodynamics and functional capacity in therapy-naive precapillary pulmonary hypertension: results from a cohort study

**DOI:** 10.1186/s12890-017-0521-4

**Published:** 2017-12-04

**Authors:** T. M. Berghaus, J. Kutsch, C. Faul, W. von Scheidt, M. Schwaiblmair

**Affiliations:** 1 Department of Cardiology, Respiratory Medicine and Intensive Care, Klinikum Augsburg, Ludwig-Maximilians-University, Munich, Germany; 2 Klinikum Augsburg, Stenglinstrasse 2, 86156 Augsburg, Germany

**Keywords:** N-terminal pro-brain-type natriuretic peptide (NT-proBNP), Pulmonary arterial hypertension (PAH), Chronic thromboembolic pulmonary hypertension (CTEPH), Exercise capacity, Hemodynamics, Age, Gender

## Abstract

**Background:**

N-terminal pro-brain-type natriuretic peptide (NT-proBNP) is currently used as a surrogate marker for disease severity in pulmonary hypertension (PH). However, NT-proBNP tends to have a high variability and may insufficiently correlate with hemodynamics and exercise capacity.

**Methods:**

To investigate the association of NT-proBNP with hemodynamics and cardio-pulmonary exercise testing (CPET) in 84 therapy-naive patients with precapillary PH.

**Results:**

NT-proBNP levels were significantly correlated with hemodynamics and CPET parameters except for cardiac index, diffusion capacity, PaO_2_ at peak exercise, and peak minute ventilation. NT-proBNP correlated best with hemodynamics and CPET in women and patients >65 years. NT-proBNP correlated better with CPET in pulmonary arterial hypertension compared to chronic thromboembolic PH (CTEPH).

**Conclusion:**

NT-proBNP is associated with disease severity in precapillary PH. The association might be age- and gender-dependent. NT-proBNP may insufficiently correlate with disease severity in CTEPH, possibly due to comorbidity.

## Background

Pulmonary hypertension (PH) is defined as an increase in mean pulmonary arterial pressure (mPAP) ≥ 25 mmHg at rest. In the presence of a pulmonary capillary wedge pressure (PCWP) ≤ 15 mmHg, PH is hemodynamically classified to be precapillary. Precapillary PH results from different clinical conditions, such as pulmonary arterial hypertension (PAH), PH due to lung diseases, chronic thromboembolic PH (CTEPH), and PH with unclear and/or multifactorial mechanisms [1].

Pressure overload of the right heart due to PH activates the natriuretic peptide system. Brain-type natriuretic peptide (BNP) is released in response to myocardial stretch from cardiomyocytes, where it has been synthesized as an inactive precursor (proBNP) and split into the active hormone BNP and the inactive N-terminal fragment (NT-proBNP) [2]. While BNP has a short half-life, NT-proBNP is not further metabolized and is eliminated only by renal excretion, resulting in a longer half-life. Therefore, NT-proBNP is preferably used in clinical routine as an indicator of myocardial dysfunction.

In PAH, serum NT-proBNP levels correlate with right heart dysfunction and provide prognostic information at the time of diagnosis and during follow-up assessments [3, 4]. However, NT-proBNP tends to have a high variability and should only be interpreted in the clinical context, as NT-proBNP may insufficiently correlate with hemodynamics and exercise capacity [1].

We therefore investigated the association of serum NT-proBNP with hemodynamics and functional capacity in therapy-naïve patients with precapillary PH.

## Methods

### Study design and patient population

Between August 2009 and March 2016, 84 patients with precapillary PH could be enrolled in the study. PH was therapy-naive in all study participants. Patients with serum creatinine levels >1.3 mg/dL and/or estimated glomerular filtration rates <50 mL/min/1.73 m^2^ or signs of acute right heart decompensation were excluded from the trial. Relevant left heart disease was ruled out by echocardiography in every patient included in the study. All laboratory tests, cardiopulmonary exercise testing (CPET), six minute walking testing (6MWT), and right heart catheterization (RHC) were performed standardised within three consecutive workdays. The study was conducted with the approval of the local Ethics Committee. Data analysis was performed retrospectively.

### Lung function tests

Pulmonary function tests included spirometry, body plethysmography, and measurement of diffusing capacity using the single-breath method (Master Screen Body and MS-PFT, Jaeger, Cardinal Health, USA). The following parameters were determined: forced vital capacity, total lung capacity, forced expiratory volume in one second, and diffusing capacity for carbon monoxide (DLCO). Blood gas analysis (ABL 725, Radiometer, Copenhagen, Denmark) was performed in arterialized capillary blood from the ear lobe without supplemental oxygen (O_2_).

### CPET

CPET was performed using a standardized protocol [5]. The work rate was continuously increased by 5–15 watts/min to a maximum tolerated level on an electromagnetically braked cycle ergometer (ViaSprint 150 p, Ergoline, Germany). Patients were encouraged to exercise until symptoms were intolerable. Blood gas analysis was done at rest and during peak exercise. The heart rate was monitored continuously and non-invasive blood pressure was taken every 2 min. The maximum work rate was recorded. O_2_ uptake (VO_2_), minute ventilation (Ve) and CO_2_ output (VCO_2_) were measured breath by breath using an adult facemask (Vmax spectra 229 D, Sensor Medics, USA). O_2_ pulse, alveolar-arterial O_2_ difference (AaDO_2_), and functional dead space ventilation (Vd/Vt) were calculated as described before [5]. The anaerobic threshold (AT) was chosen at the peak VO_2_ at which the ventilatory equivalent for O_2_ (Ve/VO_2_) increased, while the ventilatory equivalent for CO_2_ (Ve/VCO_2_) decreased or remained constant. Peak VO_2_ was defined as the value of averaged data during the final 15 s of exercise. The Ve/VCO_2_ slope was determined as the linear regression slope of Ve and VCO_2_ from the start of exercise until the respiratory compensation point (the point in time at which ventilation is stimulated by acidaemia and the end-tidal CO_2_ begins to decrease).

### RHC

RHC was performed in all patients in order to confirm precapillary PH. A thermodilution catheter (7.5 F quadruple-lumen, balloon-tipped, flow-directed, “S” Tip Swan-Ganz Catheter, Edwards Lifesciences, Irvine, USA) was inserted via the right or left femoral vein. Hemodynamic measurements were performed in supine position and included heart rate, PCWP, PAP, and right atrium pressure (RAP). O_2_ saturation was measured in mixed venous blood samples (ABL 725, Radiometer, Copenhagen, Denmark). The cardiac output was measured by thermodilution with 10 ml of sterile, ice-cold isotonic (0.9%) saline, which was injected through the right atrial lumen of the catheter; the drop in temperature at the distal thermistor was then recorded. The injectate temperature was determined by a thermistor, which was placed directly behind the right atrial inlet of the catheter. Cardiac output was calculated using a computer system (Com-2, Cardiac Output Computer, Edwards Lifesciences, Irvine, USA). In each patient, a minimum of three measurements was performed; the mean value was calculated if the variability of values was less than 10%. The pulmonary vascular resistance (PVR) was calculated using a standard formula [PVR = (mean PAP – PCWP) / cardiac output].

### NT-proBNP

Serum NT-proBNP levels were measured using a one-step sandwich chemiluminescent immunoassay (Dimension Vista™ System, Siemens Healthcare Diagnostics Inc., Newark, USA). Blood samples were taken at rest shortly before performing CPET. Values >125 pg/ml were considered elevated for patients younger than 75 years and >450 pg/ml for those older than 75 years [6]. In order to avoid an underestimation or overestimation of absolute values, measured NT-proBNP levels were divided by the age-adjusted normal upper range to calculate the normalized NT-proBNP ratio. Consequently, elevated levels result in a normalized NT-proBNP ratio > 1.

### Statistics

Statistical analysis was performed using IBM SPSS Statistics Version 23.0. Continuous variables characterized by a normal distribution are shown as means ± standard error of mean (SEM). Variables without such a distribution are expressed as medians with range. The Shapiro-Wilk test was used to check the normality of distribution. Nominal parameters were expressed as counts with percentage of total. Correlation analysis was performed using the Spearman correlation index. All results were tested for two-sided significance. *P*-values <0.05 were considered statistically significant.

## Results

### Patients’ characteristics

The characteristics of the study population are summarized in Table 1. 84 patients could be included in the trial (46 women, 38 men, mean age 70.6 years). In all subjects, precapillary pulmonary hypertension was diagnosed with a mean PAP of 41.0 mmHg and a median PVR of 7.4 Wood units. PAH was diagnosed in 75 patients, 12 study participants suffered from CTEPH. The median NT-proBNP level was 1500 pg/ml with a median NT-proBNP ratio of 7.53.

**Table 1:** Patients characteristics (*n* = 84)

Clinical profile	
Female / male [n (%)]	46 (54.8) / 38 (45.2)
Age (years)	70.6 ± 1.41
BMI (kg/m^2^)	27.1 ± 0.60
PHgroup 1 / 3 / 4 / 5 [n (%)]	63 (75) / 7 (8.3) / 12 (14.3) / 2 (2.4)
NT-proBNP	
NT-proBNP level (pg/ml)	1500 (38–13,538)
NT-proBNP ratio	7.53 (0.09–60.60)
6-min walking test	
Distance (m)	305 ± 14.3
% of norm	62.3 ± 2.72
Breaks (n)	0 (0–6)
Borg scale points (1–10)	4.10 ± 0.29
Right heart catheterization	
Mean PAP (mmHg)	41.0 ± 1.22
Cardiac output (l/min)	4.39 ± 0.14
Cardiac index (l/min/m^2^)	2.43 ± 0.10
PVR (Wood units)	7.40 (3.00–18.2)
Mean RAP (mmHg)	6.0 (1–20)
SvO_2_ (%)	61.4 ± 0.93
Lung function	
DLCO (%)	52.9 ± 2.59
PaO_2_ at rest (mmHg)	56.8 ± 1.57
PaO_2_ at peak exercise (mmHg)	57.4 ± 1.84
Cardiopulmonary exercise testing	
Work capacity (watts)	47.0 (25–150)
VO_2_ (ml/min)	953 ± 37.5
VO_2_/kg	13.0 ± 0.45
AT (ml/min/kg)	9.64 ± 0.40
O_2_ pulse at peak exercise (ml/min/beat)	8.30 (3.50–16.0)
Ve (L/min)	52.1 ± 2.11
Ve/VO_2_	40.5 ± 1.37
Ve/VCO_2_	47.2 ± 1.58
AaDO_2_ (mmHg)	49.0 ± 1.76
Vd/Vt (%)	37.1 ± 1.50
Ve/VCO_2_ slope	44.2 ± 2.06

If not stated otherwise, data is presented as mean ± SEM or as median (range)

*BMI*: body mass index, *NT-proBNP ratio*: NT-proBNP level divided by the age-adjusted normal upper range, *PAP*: pulmonary arterial pressure, *PVR*: pulmonary vascular resistance, *RAP*: right atrial pressure, *SvO*_2_: mixed venous oxygen saturation, *DLCO*: lung diffusing capacity for carbon monoxide, *PaO*_2_: arterial oxygen pressure, *VO*_2_: peak oxygen uptake, *AT*: anaerobic threshold, *Ve*: peak minute ventilation, *Ve*/*VO*_2_: oxygen equivalent at anaerobic threshold, *Ve*/*VCO*_2_: carbon dioxide equivalent at anaerobic threshold, *AaDO*_2_: alveolar-arterial oxygen difference at peak exercise, *Vd*/*Vt*: functional dead space ventilation at peak exercise, *Ve*/*VCO*_2_ slope: slope of minute ventilation to carbon dioxide output

The mean 6MWT distance was 305 m; the median work capacity 47 watts. The mean DLCO was measured to be 52.9% of predicted, the mean AaDO_2_ was 49.0 mmHg. Mean VO_2_ was determined to be 13.0 ml/min/kg with a median O_2_ pulse at peak exercise of 8.3 ml/min/beat. Mean Vd/Vt was 37.1%. The mean Ve/VO_2_ ratio was calculated to be 40.5 with a mean Ve/VCO_2_ slope of 44.2. The mean haemoglobin concentration was 14.3 ± 1.2 mg/dl with no significant differences in individual subgroups studied.

### Correlation of NT-proBNP with hemodynamics and functional capacity

In the total study population, NT-proBNP levels were significantly correlated with all parameters except CI, DLCO, PaO_2_ at peak exercise, and Ve (Table 2). Correlations were strongest for mPAP, PVR, SvO_2,_ 6MWT distance, number of breaks, work capacity, VO_2,_ O_2_ pulse, Ve/VCO_2_, AaDO_2_, and Ve/VCO_2_ slope. When adjusted for gender, correlations were more or less equal for hemodynamics and 6MWT parameters in men and women. However, correlations were stronger in females for CPET parameters, especially for work capacity and O_2_ pulse at peak exercise. When adjusted for age, NT-proBNP levels were much better correlated with hemodynamics and functional capacity in older patients than in subjects ≤65 years. NT-proBNP concentrations were more strongly correlated with exercise capacity in PAH compared to CTEPH. No relevant differences were found when NT-proBNP ratios were used instead of NT-proBNP levels (data not shown); thus, only correlations with NT-proBNP values are displayed, as they are much more established in the routine risk stratification of PH patients.

**Table 2:** Correlation of NT-proBNP with hemodynamics and functional capacity according to gender, age and PH class

		Gender		Age		PH group	
	Total	male	female	≤ 65	> 65	PAH	CTEPH
	n = 84	*n* = 38	*n* = 46	*n* = 24	*n* = 60	*n* = 63	*n* = 12
	r	r	r	r	r	r	r
6MWT							
Distance (m)	−0.430***	−0.435**	−0.395**	−0.246	−0.403**	−0.507***	−0.175
% of norm	−0.448***	−0.476**	−0.467**	−0.295	−0.486***	−0.556***	0.091
Breaks (n)	−0.497***	0.513**	0.468**	0.173	0.507***	0.556***	0.063
Borg scale	0.299**	0.283	0.340*	0.042	0.328**	0.271*	0.573
RHC							
mPAP (mmHg)	0.386***	0.454**	0.318*	0.046	0.581***	0.341**	0.900***
CO (l/min)	−0.342**	−0.338*	−0.335*	−0.495*	−0.282*	−0,322*	−0.625*
CI (l/min/m^2^)	−0.252	−0.183	−0.251	−0.261	−0.255	−0,384*	−0.100
PVR (Wood units)	0.460***	0.543**	0.458**	0.327	0.598***	0.488***	0.918***
mRAP (mmHg)	0.243*	0.255	0.231	0.045	0.270	0.215	0.260
SvO_2_ (%)	−0.527***	−0.572***	−0.491**	0.127	−0.544***	−0.530***	−0.528
Lung function							
DLCO (%)	−0.251	−0.190	−0.385*	−0.044	−0.227	−0.332*	−0.200
PaO_2_ rest (mmHg)	−0.250*	−0.240	−0.307*	−0.087	−0.237	−0.312*	0.284
PaO_2_ exercise (mmHg)	−0.166	−0.056	−0.233	−0.109	−0.142	−0.221	0.186
CPET							
Work (watts)	−0.424***	−0.280	−0.533***	−0.364	−0.401**	−0.438***	−0.173
VO_2_ (ml/min)	−0.430***	−0.479**	−0.375*	−0.417	−0.420**	−0.492***	−0.035
VO_2_/kg	−0.434***	−0.471**	−0.375*	−0.220	−0.462***	−0.514***	−0.100
AT (ml/min/kg)	−0.294*	−0.379*	−0.172	−0.319	−0.302*	−0.307	−0.618*
O_2_ pulse (ml/min/beat)	−0.409***	−0.329*	−0.497***	−0.329	−0.459***	−0.373**	−0.615*
Ve (L/min)	0.007	−0.021	0.001	−0.081	0.079	−0.024	0.119
Ve/VO_2_	0.384**	0.336	0.392*	0.791***	0.280*	0.479***	−0.091
Ve/VCO_2_	0.451***	0.351*	0.484**	0.591*	0.386**	0.465**	0.519
AaDO_2_ (mmHg)	0.398***	0.354*	0.437**	0.373	0.394**	0.452***	0.221
Vd/Vt (%)	0.351**	0.266	0.463**	0.274	0.352**	0.349**	0.460
Ve/VCO_2_ slope	0.427***	0.445**	0.433**	0.470*	0.441***	0.471***	0.671*

Level of significance: * *p* < 0.05; ** *p* < 0.01; *** *p* < 0.001

*PH*: pulmonary hypertension, *IPAH*: idiopathic pulmonary arterial hypertension, *CTEPH*: chronic thromboembolic pulmonary hypertension, 6*MWT*: 6 min walking test, *RHC*: right heart catheterisation, *PAP*: pulmonary arterial pressure,

*PVR*: pulmonary vascular resistance, *RAP*: right atrial pressure, *SvO*_2_: mixed venous oxygen saturation, *DLCO*: lung diffusing capacity for carbon monoxide,

*PaO*_2_: arterial oxygen pressure, *VO*_2_: peak oxygen uptake, *AT*: anaerobic threshold, *Ve*: peak minute ventilation, *Ve*/*VO*_2_: oxygen equivalent at anaerobic threshold,

*Ve*/*VCO*_2_: carbon dioxide equivalent at anaerobic threshold, *AaDO*_2_: alveolar-arterial oxygen difference at peak exercise, *Vd*/*Vt*: functional dead space ventilation at peak exercise, *Ve*/*VCO*_2_ slope: slope of minute ventilation to carbon dioxide output

## Discussion

Our study was conducted in order to investigate the association of serum NT-proBNP with hemodynamics and exercise capacity in therapy-naive patients with precapillary PH. NT-proBNP levels were significantly correlated with most analysed parameters. So far, our trial confirms the results of previous studies [7]. However, remarkable differences were found when correlations were adjusted for gender, age and PH class.

Gender differences in NT-proBNP levels have been described before in healthy subjects [8, 9] and in patients suffering from left heart disease [10]. In these studies, women show higher plasma concentrations of NT-proBNP [8, 10] and exhibit a greater increase with age compared to men [9]. To the best of our knowledge, no such differences have been reported in precapillary PH so far. In our trial, NT-proBNP levels were better correlated with CPET parameters in females compared to males. In general, exercise capacity is gender dependent [11], probably reflecting differences in muscle mass [12] or myocardial geometries and functions [13]. Recently, a study by *Swift* and colleagues [14] demonstrated that male PAH patients have proportionally worse right heart function despite a similar afterload compared to females. Thus, gender differences in the adaptive right ventricular remodelling in response to an impaired lung perfusion in precapillary PH might partly explain the different association of NT-proBNP and functional capacity in women and men in our trial.

**Fig. 1:**
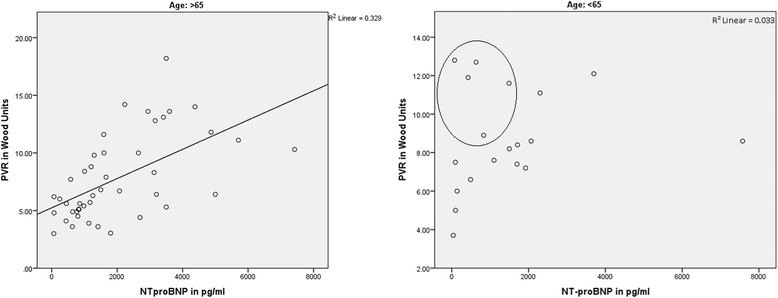
Correlation of NT-proBNP with pulmonary vascular resistance (PVR) in different age groups. Whereas older study participants show a linear correlation between the biomarker and the PVR, serum levels of NT-proBNP were much lower in younger patients, even when very high PVR values could be measured (dots in the circle)

Serum NT-proBNP concentrations increase with age in a general population, probably reflecting age-related changes in ventricular compliance, myocardial mass, or peptide clearance [8]. In contrast, functional capacity declines with age [11, 15]. Nevertheless, in our study, serum NT-proBNP levels were much better correlated with hemodynamics and functional capacity in older patients than in subjects ≤65 years, possibly reflecting a better cardio-pulmonary reserve in younger PH patients. Figure 1, for example, illustrates the relation between serum NT-proBNP levels and the PVR in different age groups. Whereas older study participants show a linear correlation between the biomarker and the PVR, serum levels of NT-proBNP were much lower in younger patients, even when very high PVR values could be measured. In general, right heart function declines with age [16]. In addition, a progressive increase in systolic PAP can be observed with advancing age even in healthy subjects [17], probably as a result of an age-associated blood vessel stiffening in the lungs. Consequently, increased afterload due to an impaired lung perfusion in precapillary PH might cause less right ventricular strain in younger individuals, possibly explaining the inferior association of serum NT-proBNP levels with disease severity in younger PH patients.

In our trial, NT-proBNP concentrations were much stronger correlated with exercise capacity in PAH compared to CTEPH. NT-proBNP indicates right ventricular pressure overload due to impaired lung perfusion in precapillary PH. For ventilation-derived CPET parameters, an impaired ventilation/perfusion matching in CTEPH compared to PAH might explain the weaker correlation with NT-proBNP in CTEPH. Ventilation/perfusion mismatching results from a more uneven lung perfusion in CTEPH, possibly due to additional thrombus formation and a more proximal vascular occlusion [18]. In contrast, PAH represents a vasculopathy that bilaterally involves distal, medium to small size arteries [19], resulting in a more balanced ventilation/perfusion match. As a consequence, ventilation-derived CPET parameters and serum NT-proBNP values might be more closely associated in PAH compared to CTEPH. However, these observations have to be interpreted with precaution. In our rather small CTEPH cohort consisting of only 12 patients, concomitant diseases, which might limit exercise capacity independently from PH, might be more prevalent than in the PAH cohort. Therefore, comorbidity might have biased our results and might yield a poorer association of NT-proBNP values with functional capacity in CTEPH patients.

We admit that our study has limitations. First, the average age of our study cohort was quite old, possibly resulting from a small proportion of “typical” idiopathic PAH patients enrolled in our trial. Advanced age could be a relevant confounder in our study. Second, only a very limited number of PH diagnostic group III and V patients could be enrolled. Thus, only PAH and CTEPH patients could be analyzed adequately. However, as only 12 CTEPH cases could be included in the analysis, results for this cohort are more speculative than for PAH patients.

## Conclusions

Despite these limitations we conclude that in therapy-naive patients with precapillary PH, serum NT-proBNP concentrations significantly correlate with disease severity. However, the association of the biomarker with hemodynamics and functional capacity might be age- and gender-dependent. In addition, NT-proBNP may insufficiently correlate with disease severity, especially in CTEPH, possibly due to the influence of comorbidity.

## Abbreviations

6MWT: 6 min walking test
AaDO_2_: Alveolar-arterial oxygen difference at peak exercise
AT: Anaerobic threshold
CPET: Cardio-pulmonary exercise testing
CTEPH: Chronic thromboembolic pulmonary hypertension
DLCO: Lung diffusing capacity for carbon monoxide
NT-proBNP: N-terminal pro-brain-type natriuretic peptide
PAH: Pulmonary arterial hypertension
PaO_2_: Arterial oxygen pressure
PAP: Pulmonary arterial pressure
PCWP: Pulmonary capillary wedge pressure
PH: Pulmonary hypertension
PVR: Pulmonary vascular resistance
RAP: Right atrial pressure
RHC: Right heart catheterization
SvO_2_: Mixed venous oxygen saturation
Vd/Vt: Functional dead space ventilation at peak exercise
Ve: Peak minute ventilation
Ve/VCO_2_ slope: Slope of minute ventilation to carbon dioxide output
Ve/VCO_2_: Carbon dioxide equivalent at anaerobic threshold
Ve/VO_2_: Oxygen equivalent at anaerobic threshold
VO_2_: Peak oxygen uptake
